# Expiratory automatic endotracheal tube compensation reduces dynamic hyperinflation in a physical lung model

**DOI:** 10.1186/cc7693

**Published:** 2009-01-23

**Authors:** Christoph Haberthür, Annekathrin Mehlig, John F Stover, Stefan Schumann, Knut Möller, Hans-Joachim Priebe, Josef Guttmann

**Affiliations:** 1 Department of Anesthesiology, Section for Surgical Intensive Care, Kantonsspital Luzern, Spitalstrasse, CH-6000 Luzern, Switzerland; 2 Department of Internal Medicine, Kantonsspital Liestal, Rheinstrasse, CH-4410 Liestal, Switzerland; 3 Division of Surgical Intensive Care Medicine, University Hospital Zurich, Rämistrasse, CH-8091 Zürich, Switzerland; 4 Department of Anesthesiology, Section for Experimental Anesthesiology, University Medical Center Freiburg, Hugstetterstrasse, D-79106 Freiburg, Germany; 5 Biomedical Engineering, University of Applied Sciences, Campus Furtwangen, Robert-Gerwig-Platz, D-78120 Furtwangen, Germany

## Abstract

**Introduction:**

The effect of expiratory endotracheal tube (ETT) resistance on dynamic lung inflation is unknown. We hypothesized that ETT resistance causes dynamic lung hyperinflation by impeding lung emptying. We further hypothesized that compensation for expiratory ETT resistance by automatic tube compensation (ATC) attenuates dynamic lung hyperinflation.

**Methods:**

A ventilator equipped with the original ATC mode and operating in a pressure-targeted mode was connected to a physical lung model that consists of four equally sized glass bottles filled with copper wool. Inspiratory pressure, peak expiratory flow, trapped lung volume and intrinsic positive end-expiratory pressure (PEEP) were assessed at combinations of four inner ETT diameters (7.0, 7.5, 8.0 and 8.5 mm), four respiratory rates (15, 20, 25 and 30/minute), three inspiratory pressures (3.0, 4.5 and 6.0 cmH_2_O) and four lung compliances (113, 86, 58 and 28 ml/cmH_2_O). Intrinsic PEEP was measured at the end of an expiratory hold manoeuvre.

**Results:**

At a given test lung compliance, inspiratory pressure and ETT size, increasing respiratory rates from 15 to 30/minutes had the following effects: inspiratory tidal volume and peak expiratory flow were decreased by means of 25% (range 0% to 51%) and 11% (8% to 12%), respectively; and trapped lung volume and intrinsic PEEP were increased by means of 25% (0% to 51%) and 26% (5% to 45%), respectively (all *P *< 0.025). At otherwise identical baseline conditions, introduction of expiratory ATC significantly attenuated (*P *< 0.025), by approximately 50%, the respiratory rate-dependent decreases in inspiratory tidal volume and the increases in trapped lung volume and intrinsic PEEP.

**Conclusions:**

In a lung model of pressure-targeted ventilation, expiratory ETT resistance caused dynamic lung hyperinflation during increases in respiratory rates, thereby reducing inspiratory tidal volume. Expiratory ATC attenuated these adverse effects.

## Introduction

In tracheally intubated and mechanically ventilated patients, expiratory resistance of an endotracheal tube (ETT) or a tracheostomy tube can cause dynamic lung hyperinflation by impeding lung emptying [1]. Dynamic hyperinflation will successively either reduce tidal volume (during some forms of pressure-targeted mechanical ventilation [2]) or increase inspiratory plateau pressure (during volume-targeted mechanical ventilation) until a new steady state is reached [3-5]. Thus far, detailed knowledge on the contribution made by the mechanical properties of the ETT to dynamic lung hyperinflation and its ventilatory consequences during pressure-targeted mechanical ventilation is lacking.

Automatic tube compensation (ATC) is an auxiliary ventilatory mode that compensates for the flow-dependent, nonlinear pressure decrease across the ETT during inspiration and expiration [6]. By facilitating lung emptying, expiratory ATC can be expected to counteract the ETT-induced dynamic lung hyperinflation. The original ATC system is equipped with a negative pressure source (generating up to 20 cmH_2_O of subatmospheric pressure). In combination with positive end-expiratory pressure (PEEP) it is used to compensate for expiratory ETT resistance. By contrast, in the commercially available ATC systems, expiratory ETT resistance is either not compensated for or its compensation is restricted to lowering the PEEP level down to zero pressure during expiration [7]. It must be emphasized that expiratory ATC is effective only when it is used in the original mode [7].

The aim of this study was to assess the ETT-related effects on dynamic lung hyperinflation during pressure-targeted mechanical ventilation in the absence and presence of expiratory tube compensation using the original ATC mode. We hypothesized that ATC would be able to counteract the ETT-related effects on dynamic lung hyperinflation. For this purpose, we used a physical lung model to simulate a wide range of ventilatory situations. In addition, we used a mathematical model of passive expiration to exclude the dynamic effects introduced by the mechanical properties of the ventilator's pneumatic components.

## Materials and methods

### Experimental set-up

**Figure 1:**
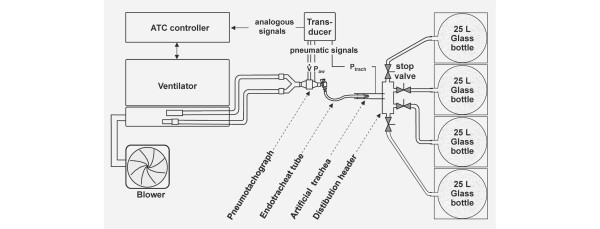
Set-up of the physical lung model. The four 25 l glass bottles were filled with copper wool to ensure isothermal volume changes. ATC, automatic tube compensation; P_aw_, airway pressure; P_trach_, tracheal pressure; $\dot{V}$, flow rate.

The experimental set-up is shown in Figure 1. The lung model is in agreement with the ISO 5369 (1987) standards. To simulate different lung compliances, four equally sized glass bottles of 25 l volume each were filled with copper wool (to ensure isothermal volume changes) and connected in parallel by a distribution header. Manual one-way valves in each of the four connecting tubes allowed ventilation of one, two, three or four bottles, resulting in approximate compliances of 25, 50, 75 and 100 ml/cmH_2_O. The distribution header was connected to an artificial trachea consisting of a transparent tube of 21 mm inner diameter (ID) [8]. The ETT under investigation was introduced into the artificial trachea. At its proximal end, the ETT was connected via a standard 15-mm bent swivel connector (Portex 100/250/001; Portex Ltd, Hythe, Kent, UK) to the Y-piece and the tubing system of an original ATC ventilator.

Theoretical concept and technical details of the original ATC ventilator, as well as differences from commercially available ATC systems, were previously reported in detail [6,7]. For the purposes of this study, the original ATC system was modified to allow expiratory hold manoeuvres. These manoeuvres were manually initiated but automatically synchronized with the ventilator's flow pattern (the manoeuvres started shortly before the beginning of the next inspiration).

Flow was measured using a Fleisch No. 2 pneumotachograph (Metabo, Epalinges, Switzerland) connected to a differential pressure transducer (CPS 1; Hoffrichter, Schwerin, Germany). Airway pressure (P_aw_) was measured via a separate opening located in the wall of the connecting diffuser of the pneumotachograph. Tracheal pressure (P_trach_) in the artificial trachea was determined via a ring channel located beyond the region of flow separation at 60 mm below the tip of the ETT [8]. P_aw _and P_trach _were measured with pressure transducers (1210A; ICSensors Inc., Milpitas, CA, USA). Flow and pressure signals were sampled at a rate of 100 Hz and digitized at 12 bits for subsequent analysis.

### Protocol

The experimental methods were approved by the local Institutional Review Board. The investigations were performed under room air (21% oxygen) during pressure-targeted mechanical ventilation using the biphasic positive airway pressure mode with an inspiratory to expiratory time ratio of 1.0, a PEEP of 5 cmH_2_O and a pressure rise time of 0.2 seconds. We alternated the upper inspiratory pressure limits at 8.0, 9.5 and 11 cmH_2_O, resulting in inspiratory driving pressures (the difference between the upper pressure limit and PEEP) of 3.0, 4.5 and 6.0 cmH_2_O, respectively. We used respiratory rates of 15, 20, 25 and 30 breaths/minute. Test lung compliances were varied by ventilating one, two, three or four of the glass bottles. ETTs (Hi-Lo Evac; Mallinckrodt Laboratories, Athlone, Ireland) of four different IDs (7.0, 7.5, 8.0, and 8.5 mm) were tested.

The different test combinations (consisting of four ETT IDs, four respiratory rates, three inspiratory pressures, and four test lung compliances) were investigated in random order, first in the absence then in the presence of ATC. We also performed the interventions in the absence of any ETT. This enabled us to assess the degree of respiratory rate-dependent dynamic hyperinflation unrelated to the mechanical properties of an ETT, and to judge the extent of ATC in absolute terms by comparing the degree of dynamic hyperinflation during ATC with that in the absence of an ETT. In these cases, the pneumotachograph was directly connected to the artificial trachea.

After steady-state conditions had been reached, the expiratory hold manoeuvre was started. The manoeuvre was terminated once P_trach _had levelled off but had been maintained for at least 10 seconds. Intrinsic PEEP was defined as peak P_trach _minus PEEP within the final 100 ms of the expiratory hold manoeuvre. Ventilatory volumes, intrinsic PEEP and other ventilatory variables were determined in the absence and presence of ATC, and in the absence of any ETT. The tidal volume generated under static conditions is referred to as the 'ideal tidal volume', and it was calculated as the product of compliance and peak inspiratory pressure. The difference between ideal and measured tidal volume constitutes the dynamically trapped volume (V_trapped.dyn_). All measured volumes were standardized to standard temperature pressure dry (STPD) conditions. Data obtained during 5 to 10 ventilatory cycles under steady-state conditions just before the start of the hold manoeuvre were stored for subsequent analysis.

Because the ventilator's expiratory valve and volume acceleration/deceleration phenomena during respiratory cycling may modify the findings to an unknown extent, we additionally assessed the effects of the various interventions in greater detail in a mathematical model of passive expiration [see Additional data file 1]. In this context, the influence of the respiratory rate and the inspiratory to expiratory time ratio was assessed by examining the expiratory cycle time (T_ex_).

### Data analysis

Differences between the test conditions – no ETT in place, ETT without ATC, and ETT with ATC (group variable) – at identical test conditions were assessed by one-way analysis of variance. If analysis of variance revealed a significant difference, then Tukey's pair-wise multiple comparisons test was performed for subsequent identification of group differences. A two-tailed *P *value below 0.025 was regarded the limit of significance. All data are presented as mean ± standard deviation.

## Results

With one, two, three or four glass bottles connected, compliances of the test lung were 29 ± 2, 58 ± 2, 86 ± 4 and 113 ± 5 ml/cmH_2_O, respectively. At these compliances, the respective calculated ideal tidal volumes varied with the inspiratory pressures above PEEP. Respective ideal tidal volumes were 88 ± 3, 172 ± 6, 257 ± 15 and 343 ± 15 ml at an inspiratory pressure of 3.0 cmH_2_O; 129 ± 7, 260 ± 10, 388 ± 13 and 509 ± 16 ml at an inspiratory pressure of 4.5 cmH_2_O; and 170 ± 11, 345 ± 13, 519 ± 16 and 674 ± 24 ml at an inspiratory pressure of 6.0 cmH_2_O. At a flow rate of 1 l/second, resistance of the test lung and the ventilator circuit (without the ETT) were constant at 5.1 cmH_2_O·s/l.

**Figure 2:**
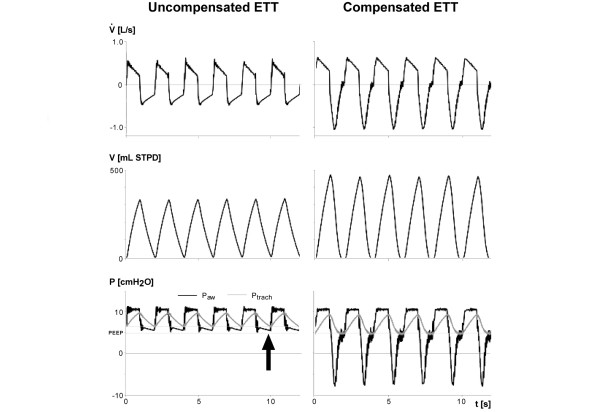
Original tracings exemplifying effects of expiratory automatic endotracheal tube compensation on lung emptying. Shown are original tracings of flow ($\dot{V}$), volume (V), airway pressure (P_aw_), and tracheal pressure (P_trach_) during pressure-targeted mechanical ventilation at a test lung compliance of 113 ml/cmH_2_O, a respiratory rate of 30/minute, and an endotracheal tube (ETT) size of 7.0 mm inner diameter without (left-hand tracings) and with (right-hand tracings) expiratory automatic tube compensation (ATC). The arrow in the left lower tracing shows that during uncompensated expiratory ETT resistance, P_trach _at the end of expiration was higher than externally applied positive end-expiratory pressure (PEEP), reflecting build-up of intrinsic PEEP because of incomplete volume emptying. During expiratory ATC, $\dot{V}$ and V increased, P_trach _at the end of expiration decreased to the level of externally applied PEEP (reflecting absence of intrinsic PEEP), and peak pressure remained unchanged. These findings indicate facilitated volume emptying during expiratory ATC. STPD: standard temperature pressure dry.

The principal effects of ATC are captured in original tracings of airflow, volume and airway and tracheal pressures (Figure 2). Compared with no ATC, compensation for expiratory ETT resistance by ATC increased tidal volume and decreased P_trach _to the level of external PEEP, reflecting reduced intrinsic PEEP and therefore reduced dynamic hyperinflation. ATC did not affect peak P_trach_, peak inspiratory pressure or PEEP.

**Figure 3:**
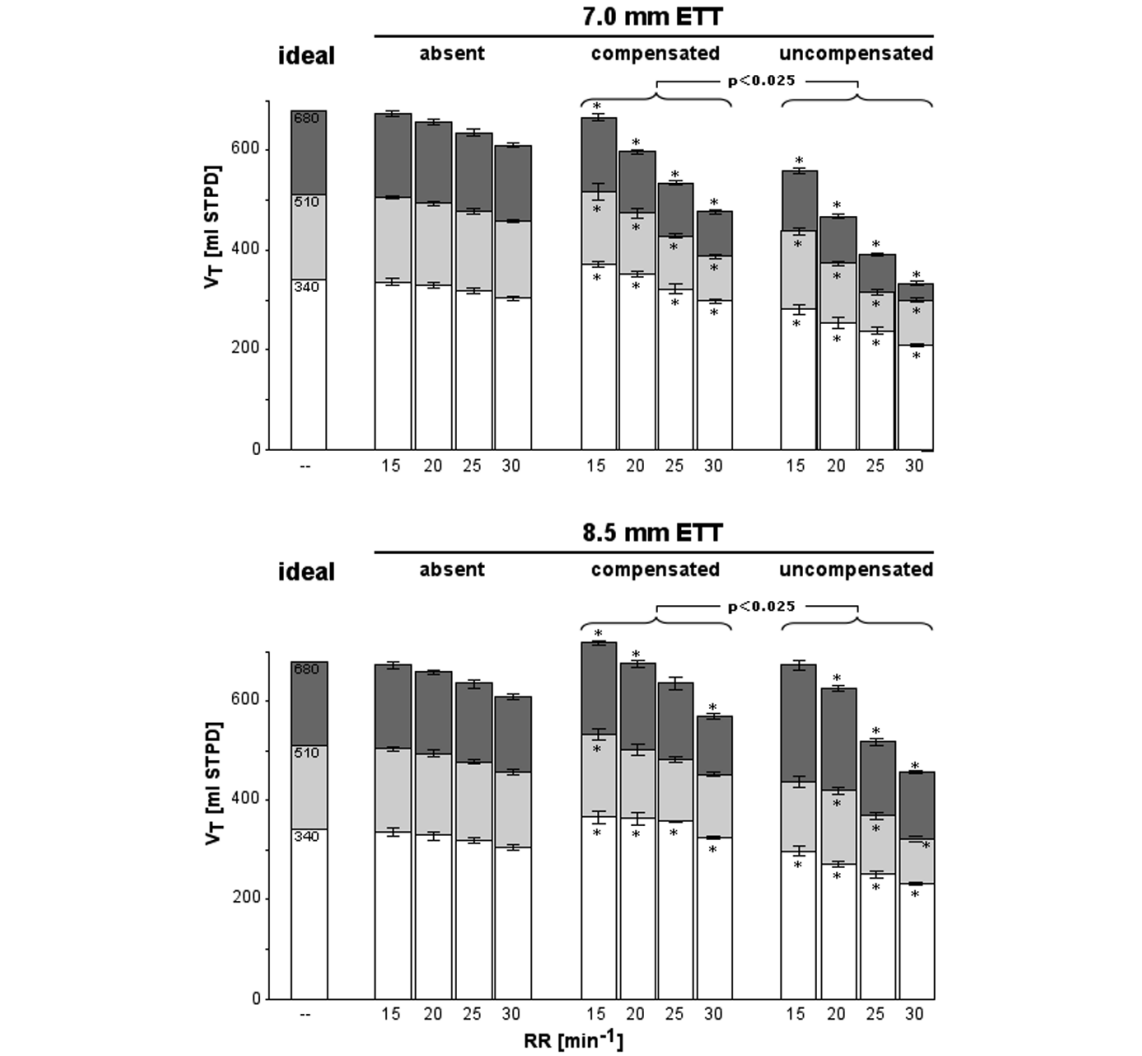
Results from the physical lung model. Shown is the effect of increasing respiratory rates (RRs) on tidal volumes (V_T_s) in the absence of any endotracheal tube (ETT) and in the presence of an ETT with (compensated) and without (uncompensated) expiratory automatic tube compensation at a test lung compliance of 113 ml/cmH_2_O, ETT sizes of 7.0 mm (top) and 8.5 mm inner diameter (bottom), and inspiratory pressures of 3.0 (white bars), 4.5 (light grey bars) and 6.0 cmH_2_O (dark grey bars). 'Ideal' indicates tidal volume calculated on the basis of peak inspiratory pressure and compliance. Bars and whiskers indicate means ± standard deviation. Note that at low RRs, V_T_s during expiratory automatic tube compensation exceeded those observed in the absence of any ETT, particularly at the larger ETT size. *P < 0.025 versus absent ETT. STPD: standard temperature pressure dry.

At ETT sizes of 7.0 and 8.5 mm ID, the respiratory rate-induced decreases in tidal volume were significantly less in the presence than in the absence of expiratory ATC (*P *< 0.025; Figure 3). Findings were comparable for ETT sizes 7.5 and 8.0 mm ID (data not shown). In agreement with these findings, when increasing respiratory rates from 15 to 30/minute at a lung compliance of 113 ml/cmH_2_O, an ETT size of 7.0 mm ID and peak inspiratory pressures of 3.0, 4.5 and 6.0 cmH_2_O, the corresponding peak expiratory flows were approximately twice as high, and intrinsic PEEP and dynamically trapped volume were approximately twice as low in the presence compared with the absence of expiratory ATC (Table 1). Under otherwise identical conditions, the effects of ATC on the various ventilatory variables were qualitatively similar but increasingly less pronounced at lung compliances of 89 and 58 ml/cmH_2_O. At a test lung compliance of 29 ml/cmH_2_O, ATC did not exhibit any significant effect (data not shown).

**Table 1:** Peak expiratory flow, intrinsic PEEP and trapped volume

RR (l/minute)	Without ETT			Compensated ETT			Uncompensated ETT		
			
	${\dot{V}}_{ex.\max}$ (l/second)	Intrinsic PEEP (cmH_2_O)	V_trapped.dyn _(ml)	${\dot{V}}_{ex.\max}$ (l/second)	Intrinsic PEEP (cmH_2_O)	V_trapped.dyn _(ml)	${\dot{V}}_{ex.\max}$ (l/second)	Intrinsic PEEP (cmH_2_O)	V_trapped.dyn _(ml)
Inspiratory pressure of 3.0 cmH_2_O									
15	0.75 ± 0.00	0.15 ± 0.02	4 ± 2	0.96 ± 0.02	0.15 ± 0.03	-31 ± 3	0.37 ± 0.02	0.79 ± 0.08	58 ± 8
20	0.74 ± 0.02	0.16 ± 0.01	12 ± 4	0.92 ± 0.01	0.15 ± 0.01	-12 ± 5	0.36 ± 0.02	0.88 ± 0.06	58 ± 9
25	0.70 ± 0.01	0.21 ± 0.04	23 ± 2	0.85 ± 0.01	0.29 ± 0.03	18 ± 10	0.36 ± 0.01	0.82 ± 0.02	77 ± 6
30	0.69 ± 0.01	0.29 ± 0.02	36 ± 2	0.77 ± 0.01	0.32 ± 0.02	43 ± 5	0.34 ± 0.01	0.97 ± 0.03	109 ± 2
Inspiratory pressure of 4.5 cmH_2_O									
15	1.12 ± 0.01	0.18 ± 0.03	6 ± 1	1.17 ± 0.01	0.18 ± 0.01	-5 ± 30	0.48 ± 0.01	0.63 ± 0.02	54 ± 5
20	1.10 ± 0.01	0.22 ± 0.03	18 ± 6	1.13 ± 0.01	0.38 ± 0.00	37 ± 11	0.46 ± 0.01	0.93 ± 0.00	96 ± 2
25	1.08 ± 0.01	0.30 ± 0.02	34 ± 2	1.06 ± 0.01	0.42 ± 0.02	82 ± 2	0.45 ± 0.01	1.28 ± 0.02	160 ± 3
30	1.02 ± 0.01	0.49 ± 0.02	54 ± 4	0.97 ± 0.02	0.50 ± 0.02	124 ± 3	0.43 ± 0.01	1.48 ± 0.01	240 ± 3
Inspiratory pressure of 6.0 cmH_2_O									
15	1.50 ± 0.04	0.16 ± 0.02	8 ± 2	1.22 ± 0.01	0.19 ± 0.01	15 ± 6	0.57 ± 0.01	0.91 ± 0.02	60 ± 5
20	1.45 ± 0.01	0.21 ± 0.02	25 ± 2	1.20 ± 0.01	0.38 ± 0.01	84 ± 2	0.55 ± 0.01	1.38 ± 0.02	161 ± 2
25	1.43 ± 0.02	0.40 ± 0.01	46 ± 5	1.18 ± 0.01	0.52 ± 0.01	147 ± 3	0.52 ± 0.01	1.71 ± 0.01	246 ± 1
30	1.39 ± 0.02	0.63 ± 0.01	72 ± 2	1.13 ± 0.01	0.56 ± 0.02	204 ± 4	0.50 ± 0.01	2.03 ± 0.01	310 ± 1

Shown are the peak expiratory flow, intrinsic PEEP and trapped volume at a test lung compliance of 113 ml/cmH_2_O and an ETT of 7.0 mm ID. Values are expressed as mean ± standard deviation. Respective differences between uncompensated and compensated ETT are significantly different (*P *< 0.025). ATC, automatic tube compensation; ETT, endotracheal tube; ID, inner diameter; PEEP, positive end-expiratory pressure; RR, respiratory rate; ${\dot{V}}_{ex.\max}$, peak expiratory flow; V_trapped.dyn_, trapped lung volume (difference between calculated [ideal] and measured inspiratory lung volume).

During expiratory ATC at the lower respiratory rates and inspiratory pressures, actual tidal volumes were slightly but significantly (*P *< 0.025) larger than the calculated ideal tidal volumes (maximally 31 and 37 ml with the use of ETTs of 7.0 mm and 8.5 mm ID, respectively; Figure 3).

**Figure 4:**
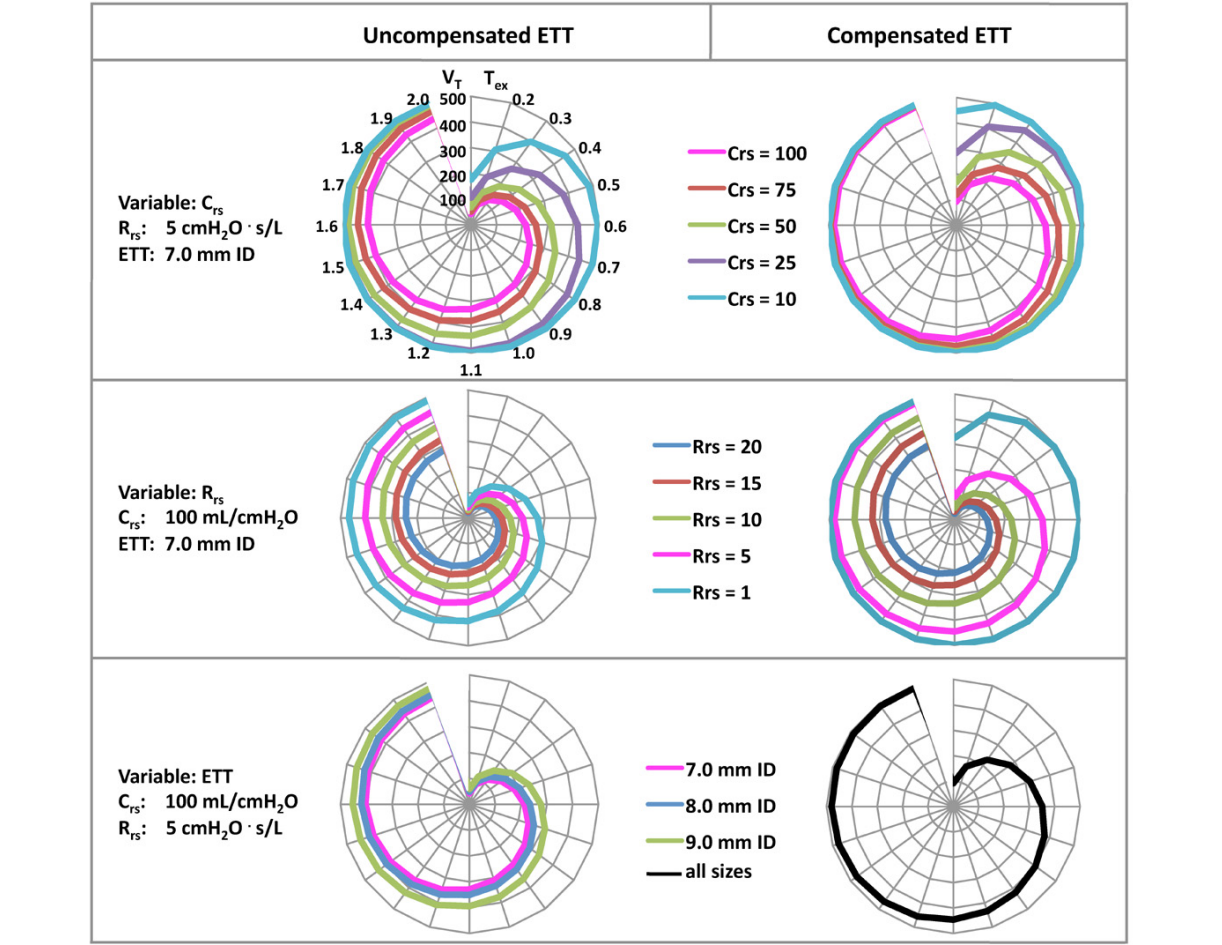
Results from the mathematical lung model. Shown is the influence of respiratory system compliance (C_rs_; top), respiratory system resistance (R_rs_; middle) and size of endotracheal tube (ETT; bottom) on tidal volume (V_T_; ml STPD) at different expiratory cycle times (T_ex_; ms) during uncompensated ETT (left hand diagrams) or ideally compensated ETT (right hand diagrams). Values are derived from the mathematical model in which ventilation was simulated with pressure-targeted ventilation aimed at generating a tidal volume of 500 ml throughout (outer circles in the net diagrams). The larger the area within the coloured curve, the greater V_T _becomes (ideally 500 ml), reflecting a decrease in the dynamically trapped volume. For all diagrams, units of V_T _and T_ex _are depicted in the left-hand top diagram. ID, inner diameter.

Computation in the mathematical model showed that at a given expiratory cycle time (T_ex_) reduction in tidal volume (as an indicator of dynamic hyperinflation) must be expected to worsen with increasing compliance, increasing resistance and decreasing ID of the ETT (Figure 4). This adverse effect on tidal volume can be expected to be attenuated by expiratory ATC.

## Discussion

The main findings of this *in vitro *investigation can be summarized as follows. First, with an ETT in place, tidal volumes decreased, and intrinsic PEEP and trapped volume increased in a respiratory rate-dependent manner under all experimental conditions. Second, expiratory ATC blunted the respiratory rate-dependent decreases in tidal volume, and the increases in intrinsic PEEP and trapped volume at all three inspiratory pressures, four ETT internal diameters and at three of the four compliances studied. Finally, the efficacy of expiratory ATC decreased with decreasing compliances. In agreement with these findings in the physical lung model, the mathematical model suggests that efficacy of expiratory ATC can be expected to be highest at normal to high lung compliance, low airway resistance and small ETT size. The findings confirm our hypothesis that expiratory ATC would counteract ETT-related adverse effects on dynamic lung hyperinflation.

Pulmonary hyperinflation is defined as an increase in the functional residual capacity above normal [9]. It can be quantified in terms of volume (trapped volume) or pressure (intrinsic PEEP). Various conditions can promote pulmonary hyperinflation, most of them being related to the mechanical properties of the patient's respiratory system and/or the ventilator settings. For example, pulmonary hyperinflation may result from loss of lung recoil (as in emphysema) and/or dynamic hyperinflation. The latter, in turn, may be caused by reduced expiratory flow (as with increased airway resistance) and/or insufficient duration of expiration (T_ex_) required for complete lung emptying. T_ex_, in turn, depends on respiratory rate and inspiratory to expiratory time ratio. Furthermore, lung emptying also depends on the preceding inspiratory volume, and thus on inspiratory driving pressure and the respiratory system's compliance.

To our knowledge, this is the first study that focuses on the effect of expiratory ETT resistance on dynamic hyperinflation and its ventilatory consequences during pressure-targeted mechanical ventilation. This is of considerable clinical relevance because the ETT-related ventilatory effects during mechanical ventilation are anything but trivial, with up to 50% reduction in tidal volume due to expiratory ETT resistance, as demonstrated in the present investigation. Our findings suggest that expiratory ETT resistance may cause clinically relevant dynamic lung hyperinflation by impeding lung emptying. These ETT-related problems worsened with increasing respiratory rate, inspiratory to expiratory time ratio and lung compliance, and with decreasing airway resistance and ID of the ETT. We could demonstrate that the ETT-induced adverse ventilatory effects can be significantly attenuated if expiratory ETT resistance is compensated for by expiratory ATC. This intervention was most effective at normal to high lung compliance, low airway resistance and small ETT size. Our findings suggest that in addition to the known favourable effects of ATC in the spontaneously breathing patient [10-13], the ATC mode may also be advantageous in mechanically ventilated patients.

An expiratory support system using a computer-controlled piston pump as a negative pressure source to augment exhalation was recently proposed to avoid air trapping [14]. This system works by matching expiratory (aspirated) and inspiratory (delivered) volumes, aiming to preserve the exponential shape of the expiratory flow pattern by using the entire duration of expiration. However, it requires knowledge of current mechanical properties (respiratory system compliance, respiratory system resistance, and resistance of the ETT). Because the system is designed to counteract increased expiratory resistance irrespective of its origin (caused by expiratory ETT resistance and/or airway resistance), subatmospheric pressure will be transmitted not only to the artificial airway but also to the lower airways. This carries a risk for airway collapse in susceptible patients (for instance, in those suffering from exacerbated chronic obstructive pulmonary disease). Furthermore, because the system works on matching expiratory and inspiratory volumes, an air leak may cause overcompensation and thus can lead to an inadequate fall in tracheal and possibly even in alveolar pressure. Thus far, the system has only been bench tested. By contrast, the ATC system has been extensively tested in humans. In addition, because compensation is targeted at ETT resistance and not at airway resistance, the ATC mode prevents P_trach _from decreasing below the preset PEEP level [7] (Figure 2). Furthermore, knowledge of current respiratory system compliance and resistance is not required, because ATC is based on P_trach_, which is derived from the ETT-specific coefficients and the continuously measured P_aw _and airflow rate [6,8].

### Clinical implications

Our findings indicate that the detrimental effects of expiratory ETT resistance on lung emptying should become clinically relevant with increasing compliance, decreasing airway resistance, decreasing ETT size and decreasing T_ex _(the latter being a function of respiratory rate and inspiratory to expiratory time ratio). The combination of high respiratory rates and short expiratory times is observed in patients with the acute respiratory distress syndrome (ARDS). If these patients are ventilated with high respiratory rates and tidal volumes (or peak inspiratory pressures) at the upper level of acceptance [15], then T_ex _may become too short to allow complete lung emptying despite high expiratory flow rates, because of low lung compliance in ARDS. In such situation, compensation for expiratory ETT resistance by expiratory ATC may be a suitable means of facilitating lung emptying. Whereas our mathematical model would support such an assumption in general (Figure 4, upper panels), the physical lung model study showed a lack of efficacy of expiratory ATC at very low lung compliances. This apparent contradiction is best explained by the finite efficacy of real compared with ideal expiratory ETT compensation [7].

The problems associated with ETT-induced increased expiratory resistance are not necessarily restricted to patients with diseased lungs during controlled ventilation, but can equally be expected in patients with normal lung mechanics requiring high respiratory rates (for instance, because of increased ventilatory demand), as may be the case during assisted spontaneous breathing (for example, during pressure support ventilation). The high respiratory rates then will result in a close to 1:1 inspiratory to expiratory time ratio. In such cases, dynamic lung hyperinflation may be more the rule than the exception. Incomplete lung emptying during pressure support ventilation will increase inspiratory airway pressures (rather than decrease tidal volume as during pressure-targeted mechanical ventilation), possibly leading to patient-ventilator asynchrony by futile inspiratory efforts [16].

At first glance, patients with exacerbated chronic obstructive pulmonary disease (COPD) should also benefit from expiratory ATC. However, increased airway resistance in such patients will mostly exceed the resistive properties of the ETT. Based on this and on our findings in the mathematical model (Figure 4, middle panels), it is therefore unlikely that such patients will benefit from expiratory ATC.

### Limitations

This study carries the obvious limitations of any bench study. The data were acquired in physical and mathematical lung models. Measurements were made at constant compliances, thereby excluding intratidal nonlinearity of compliance, which may be present in patients with severe pulmonary disease. Similarly, the effect of regional imbalances in lung mechanics with parallel heterogeneity in time constants and airway stiffness could not be assessed. Thus, caution must be exercised when applying the findings to clinical practice.

Statistically significant but clinically negligible increases in tidal volume above those denoted as ideal were observed during expiratory ATC, predominantly at low respiratory rates and small tidal volumes. They reflect a slight overshoot of the ATC controller during tight ETT compensation, which is, however, of little if any clinical relevance [7,13].

Differences in the effects of the various interventions on ventilatory variables in the presence (with expiratory ATC) of an ETT and in the absence (by necessity without expiratory ATC) of an ETT indicate that, although expiratory ATC clearly attenuated the adverse effects of expiratory ETT resistance, it is (not yet) able to counteract this resistance fully [7,10].

## Conclusion

High expiratory ETT resistance can cause dynamic hyperinflation, which in turn will reduce inspiratory tidal volume under most forms of pressure-targeted mechanical ventilation. These ETT-related side effects are markedly attenuated by expiratory ATC.

## Key messages

• Resistance of the ETT during expiration can markedly impede lung emptying, worsening with increasing respiratory rate and lung compliance, and with decreasing airway resistance and ETT size.

• Under most forms of pressure-targeted mechanical ventilation, the ETT-related effects on lung emptying are inevitably associated with reduction in the inspiratory tidal volume of up to 50%.

• Compensation for expiratory tube resistance by the (original) ATC mode is a highly attractive way to minimize ETT-related impairment of lung emptying.

• In contrast to existing commercially available ATC systems, only the original ATC system is equipped with a negative pressure source that, in combination with PEEP, compensates for expiratory ETT resistance.

## Abbreviations

ATC: automatic tube compensation; ETT: endotracheal tube; ID: inner diameter; P_aw_: airway pressure; PEEP: positive end-expiratory pressure; P_trach_: tracheal pressure; T_ex_: expiratory cycle time.

## Competing interests

The authors declare that they have no competing interests.

## Authors' contributions

CH designed the study and drafted the manuscript. AM performed measurements in the physical lung model. JS and HJP participated in manuscript drafting. SS ran analyses on the mathematical lung model. KM performed statistical analysis. JG designed the mathematical lung model. All authors read and approved the final manuscript.

## Supplementary Material

Additional file 1 Word document providing a detailed description of the mathematical lung model for passive expiration. Click here for file
