# Supplementary material for: Expiratory automatic endotracheal tube compensation reduces dynamic hyperinflation in a physical lung model
Source: Crit Care. 2009 Jan 23;13(1):R4. doi: 10.1186/cc7693 (PMC2688116; doi:10.1186/cc7693)
Supplement: Additional file 1 — Word document providing a detailed description of the mathematical lung model for passive expiration. [file cc7693-S1.doc]

**Mathematical lung model**

## In the mathematical model study we used a mathematical description of the flow curve during passive expiration for uncompensated and compensated airflow resistance of the endotracheal tube (ETT). During passive expiration the driving pressure which results from the volume-loaded compliance at the end of the preceding inspiration is used to overcome the sum of pressure decreases across the resistance of the respiratory system (PR_rs_), the resistance of the ETT (PR_ETT_), and the resistance of the expiratory limb (PR_EX_):

|1|

According to the equation of motion and Rohrer’s approach we can replace as follows:

|2a|

|2b|

|2c|

whereby $\dot{V}_{\text{ex}}$is expiratory flow rate, and k_1_ and k_2_ are the ETT specific coefficients. By inserting equations |2a|, |2b| and |2c| into equation |1| we get:

|3|

Equation |3| has the form of a second degree polynomial for $\dot{V}_{\text{ex}}$with the following solution:

|4|

If the ETT resistance is ideally (fully) compensated, equation |3| can be simplified as follows:

|5|

Following rearrangement of equation |5| we get:

|6|

Equations |4| and |6| were programmed in a MS Excel® worksheet (available upon request from the corresponding author). Simulation was performed at the same tidal volumes or inspiratory pressures, respiratory rates, and ETT sizes (i.e., identical tube coefficients k_1_ and k_2_), and the same values for C_rs_, R_rs_, R_EX_ as those used or determined in the physical model.
